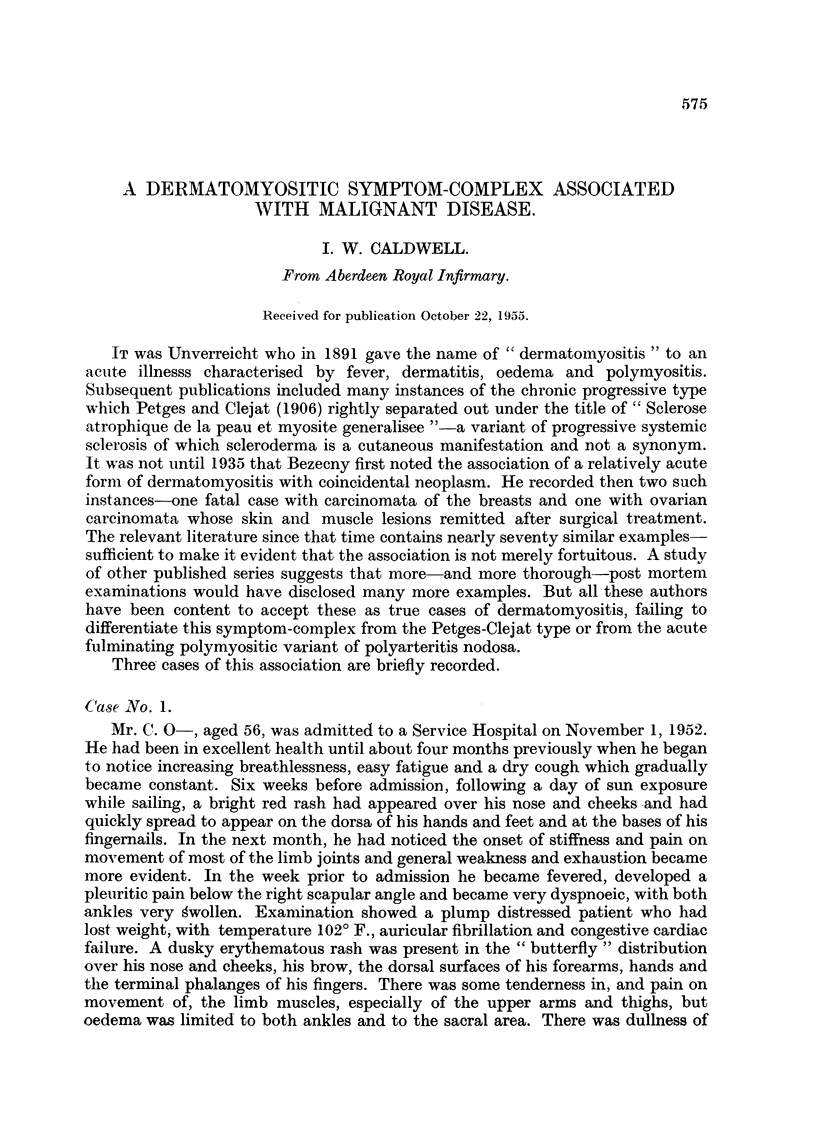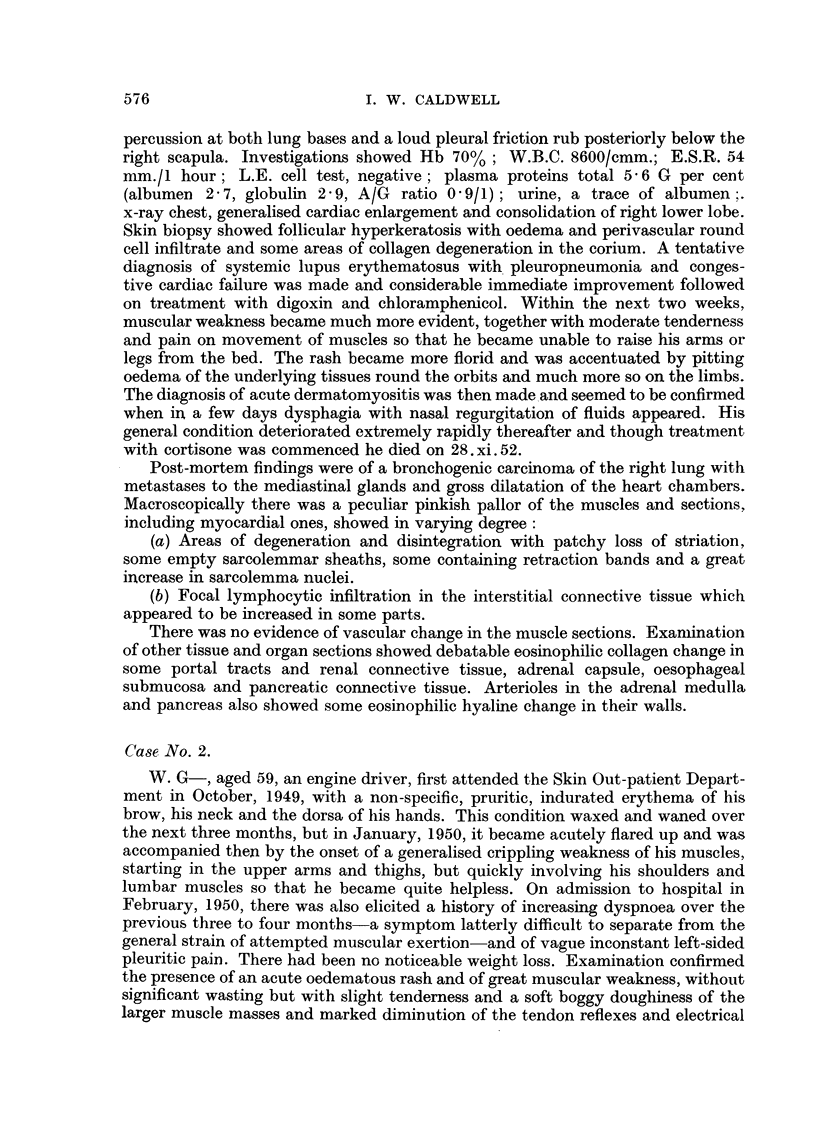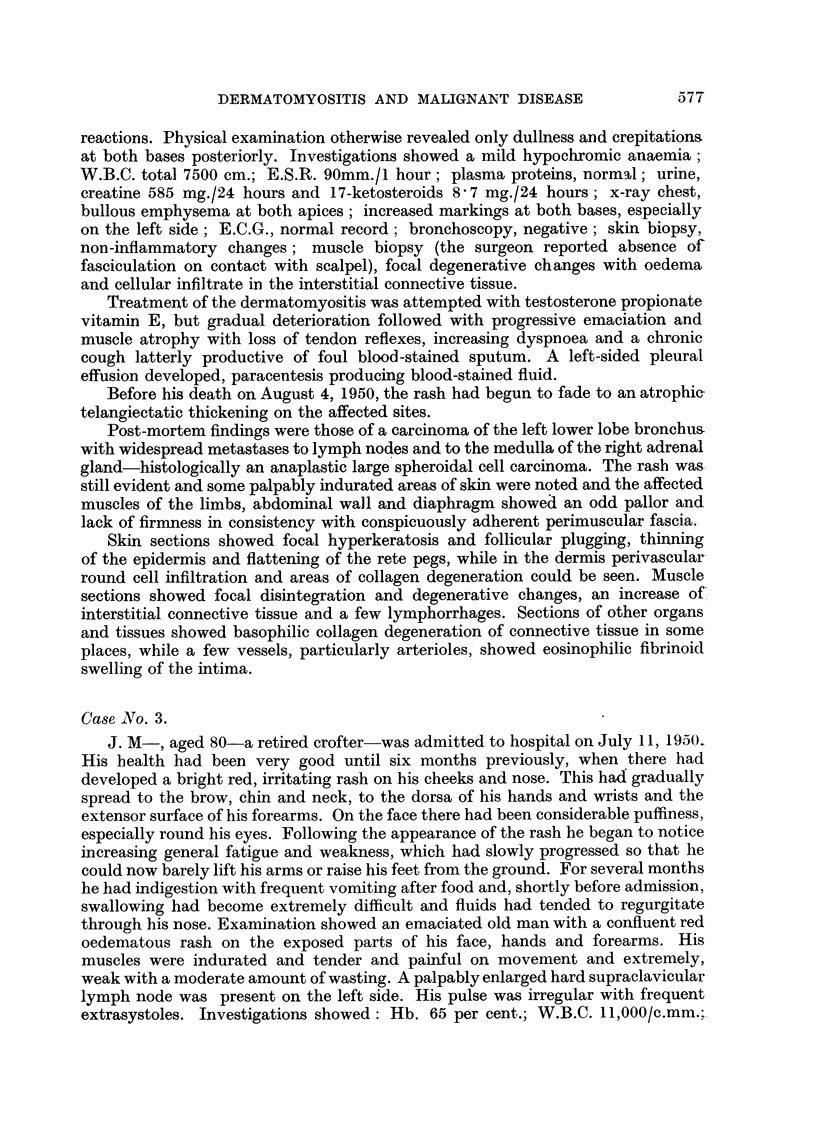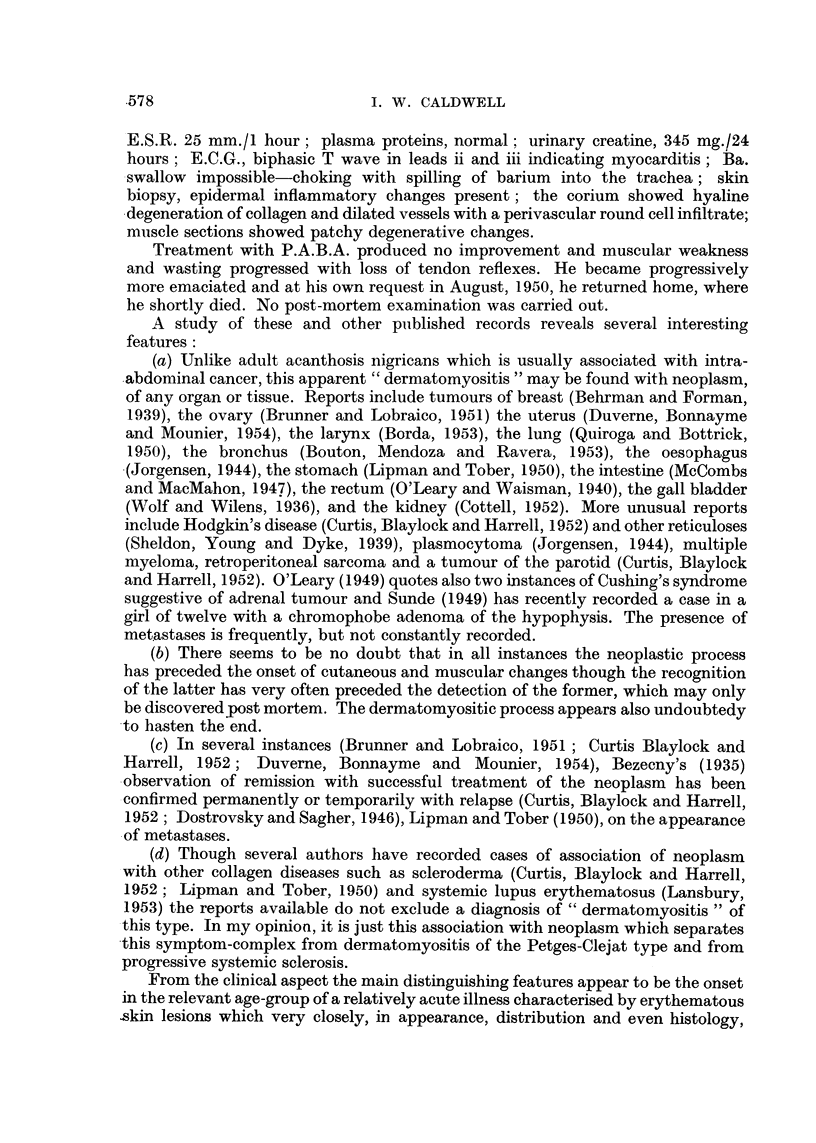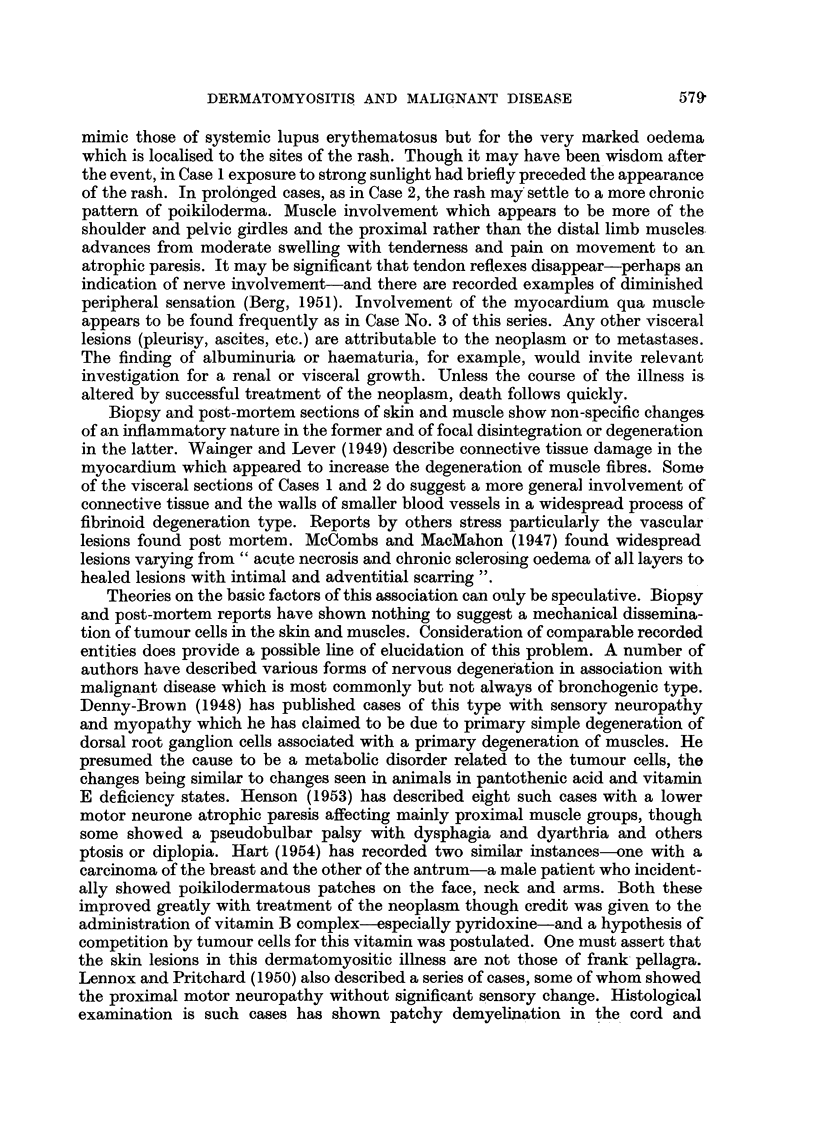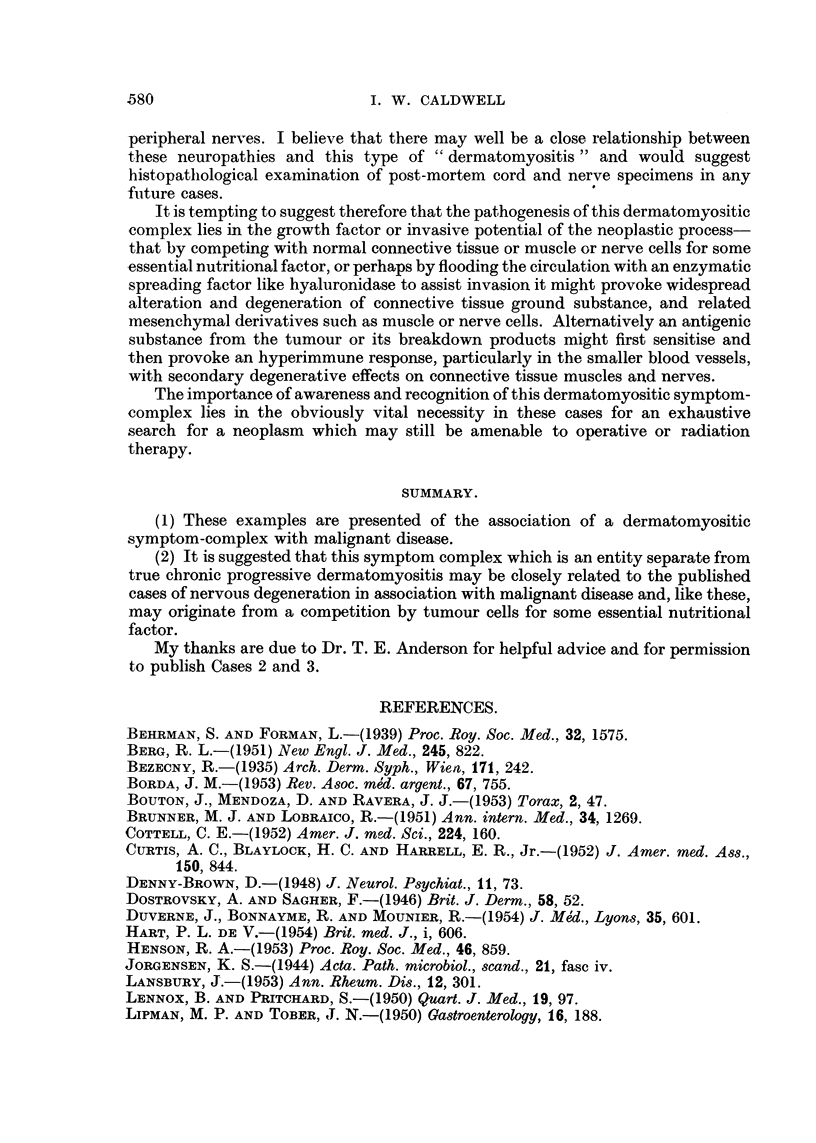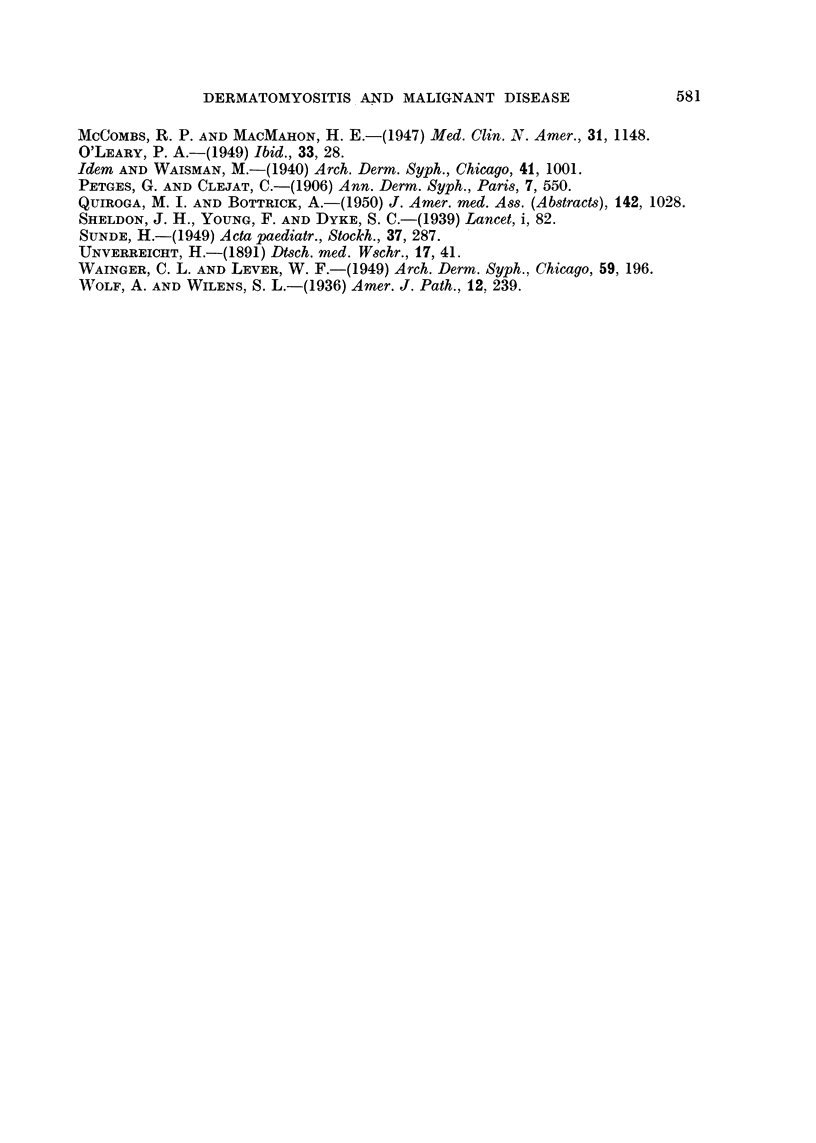# A Dermatomyositic Symptom-Complex Associated with Malignant Disease

**DOI:** 10.1038/bjc.1955.58

**Published:** 1955-12

**Authors:** I. W. Caldwell


### A DERMATOMYOSITIC SYMPTOM-COMPLEX ASSOCIATED WITH MALIGNANT DISEASE.

I. W. CALDWELL.

*From Aberdeen Royal Infirmary.*

Received for publication October 22, 1955.

IT was Unverreicht who in 1891 gave the name of " dermatomyositis " to an acute illnesss characterised by fever, dermatitis, oedema and polymyositis. Subsequent publications included many instances of the chronic progressive type which Petges and Clejat (1906) rightly separated out under the title of " Sclerose atrophique de la peau et myosite generalisee "—a variant of progressive systemic sclerosis of which scleroderma is a cutaneous manifestation and not a synonym. It was not until 1935 that Bezecny first noted the association of a relatively acute form of dermatomyositis with coincidental neoplasm. He recorded then two such instances—one fatal case with carcinomata of the breasts and one with ovarian carcinomata whose skin and muscle lesions remitted after surgical treatment. The relevant literature since that time contains nearly seventy similar examples—sufficient to make it evident that the association is not merely fortuitous. A study of other published series suggests that more—and more thorough—post mortem examinations would have disclosed many more examples. But all these authors have been content to accept these as true cases of dermatomyositis, failing to differentiate this symptom-complex from the Petges-Clejat type or from the acute fulminating polymyositic variant of polyarteritis nodosa.

Three cases of this association are briefly recorded.

*Case No.* 1.

Mr. C. O—, aged 56, was admitted to a Service Hospital on November 1, 1952. He had been in excellent health until about four months previously when he began to notice increasing breathlessness, easy fatigue and a dry cough which gradually became constant. Six weeks before admission, following a day of sun exposure while sailing, a bright red rash had appeared over his nose and cheeks and had quickly spread to appear on the dorsa of his hands and feet and at the bases of his fingernails. In the next month, he had noticed the onset of stiffness and pain on movement of most of the limb joints and general weakness and exhaustion became more evident. In the week prior to admission he became fevered, developed a pleuritic pain below the right scapular angle and became very dyspnoeic, with both ankles very swollen. Examination showed a plump distressed patient who had lost weight, with temperature 102° F., auricular fibrillation and congestive cardiac failure. A dusky erythematous rash was present in the " butterfly " distribution over his nose and cheeks, his brow, the dorsal surfaces of his forearms, hands and the terminal phalanges of his fingers. There was some tenderness in, and pain on movement of, the limb muscles, especially of the upper arms and thighs, but oedema was limited to both ankles and to the sacral area. There was dullness of percussion at both lung bases and a loud pleural friction rub posteriorly below the right scapula. Investigations showed Hb 70% ; W.B.C. 8600/cmm.; E.S.R. 54 mm./1 hour ; L.E. cell test, negative ; plasma proteins total 5·6 G per cent (albumen 2·7, globulin 2·9, A/G ratio 0·9/1) ; urine, a trace of albumen ;. x-ray chest, generalised cardiac enlargement and consolidation of right lower lobe. Skin biopsy showed follicular hyperkeratosis with oedema and perivascular round cell infiltrate and some areas of collagen degeneration in the corium. A tentative diagnosis of systemic lupus erythematosus with pleuropneumonia and congestive cardiac failure was made and considerable immediate improvement followed on treatment with digoxin and chloramphenicol. Within the next two weeks, muscular weakness became much more evident, together with moderate tenderness and pain on movement of muscles so that he became unable to raise his arms or legs from the bed. The rash became more florid and was accentuated by pitting oedema of the underlying tissues round the orbits and much more so on the limbs. The diagnosis of acute dermatomyositis was then made and seemed to be confirmed when in a few days dysphagia with nasal regurgitation of fluids appeared. His general condition deteriorated extremely rapidly thereafter and though treatment with cortisone was commenced he died on 28.xi.52.

Post-mortem findings were of a bronchogenic carcinoma of the right lung with metastases to the mediastinal glands and gross dilatation of the heart chambers. Macroscopically there was a peculiar pinkish pallor of the muscles and sections, including myocardial ones, showed in varying degree :

(*a*) Areas of degeneration and disintegration with patchy loss of striation, some empty sarcolemmar sheaths, some containing retraction bands and a great increase in sarcolemma nuclei.

(*b*) Focal lymphocytic infiltration in the interstitial connective tissue which appeared to be increased in some parts.

There was no evidence of vascular change in the muscle sections. Examination of other tissue and organ sections showed debatable eosinophilic collagen change in some portal tracts and renal connective tissue, adrenal capsule, oesophageal submucosa and pancreatic connective tissue. Arterioles in the adrenal medulla and pancreas also showed some eosinophilic hyaline change in their walls.

*Case No.* 2.

W. G—, aged 59, an engine driver, first attended the Skin Out-patient Department in October, 1949, with a non-specific, pruritic, indurated erythema of his brow, his neck and the dorsa of his hands. This condition waxed and waned over the next three months, but in January, 1950, it became acutely flared up and was accompanied then by the onset of a generalised crippling weakness of his muscles, starting in the upper arms and thighs, but quickly involving his shoulders and lumbar muscles so that he became quite helpless. On admission to hospital in February, 1950, there was also elicited a history of increasing dyspnoea over the previous three to four months—a symptom latterly difficult to separate from the general strain of attempted muscular exertion—and of vague inconstant left-sided pleuritic pain. There had been no noticeable weight loss. Examination confirmed the presence of an acute oedematous rash and of great muscular weakness, without significant wasting but with slight tenderness and a soft boggy doughiness of the larger muscle masses and marked diminution of the tendon reflexes and electrical reactions. Physical examination otherwise revealed only dullness and crepitations at both bases posteriorly. Investigations showed a mild hypochromic anaemia ; W.B.C. total 7500 cm.; E.S.R. 90mm./1 hour ; plasma proteins, normal; urine, creatine 585 mg./24 hours and 17-ketosteroids 8·7 mg./24 hours ; x-ray chest, bullous emphysema at both apices ; increased markings at both bases, especially on the left side ; E.C.G., normal record ; bronchoscopy, negative ; skin biopsy, non-inflammatory changes ; muscle biopsy (the surgeon reported absence of fasciculation on contact with scalpel), focal degenerative changes with oedema and cellular infiltrate in the interstitial connective tissue.

Treatment of the dermatomyositis was attempted with testosterone propionate vitamin E, but gradual deterioration followed with progressive emaciation and muscle atrophy with loss of tendon reflexes, increasing dyspnoea and a chronic cough latterly productive of foul blood-stained sputum. A left-sided pleural effusion developed, paracentesis producing blood-stained fluid.

Before his death on August 4, 1950, the rash had begun to fade to an atrophic telangiectatic thickening on the affected sites.

Post-mortem findings were those of a carcinoma of the left lower lobe bronchus with widespread metastases to lymph nodes and to the medulla of the right adrenal gland—histologically an anaplastic large spheroidal cell carcinoma. The rash was still evident and some palpably indurated areas of skin were noted and the affected muscles of the limbs, abdominal wall and diaphragm showed an odd pallor and lack of firmness in consistency with conspicuously adherent perimuscular fascia.

Skin sections showed focal hyperkeratosis and follicular plugging, thinning of the epidermis and flattening of the rete pegs, while in the dermis perivascular round cell infiltration and areas of collagen degeneration could be seen. Muscle sections showed focal disintegration and degenerative changes, an increase of interstitial connective tissue and a few lymphorrhages. Sections of other organs and tissues showed basophilic collagen degeneration of connective tissue in some places, while a few vessels, particularly arterioles, showed eosinophilic fibrinoid swelling of the intima.

*Case No.* 3.

J. M—, aged 80—a retired crofter—was admitted to hospital on July 11, 1950. His health had been very good until six months previously, when there had developed a bright red, irritating rash on his cheeks and nose. This had gradually spread to the brow, chin and neck, to the dorsa of his hands and wrists and the extensor surface of his forearms. On the face there had been considerable puffiness, especially round his eyes. Following the appearance of the rash he began to notice increasing general fatigue and weakness, which had slowly progressed so that he could now barely lift his arms or raise his feet from the ground. For several months he had indigestion with frequent vomiting after food and, shortly before admission, swallowing had become extremely difficult and fluids had tended to regurgitate through his nose. Examination showed an emaciated old man with a confluent red oedematous rash on the exposed parts of his face, hands and forearms. His muscles were indurated and tender and painful on movement and extremely, weak with a moderate amount of wasting. A palpably enlarged hard supraclavicular lymph node was present on the left side. His pulse was irregular with frequent extrasystoles. Investigations showed : Hb. 65 per cent.; W.B.C. 11,000/c.mm.;

E.S.R. 25 mm./1 hour ; plasma proteins, normal ; urinary creatine, 345 mg./24 hours ; E.C.G., biphasic T wave in leads ii and iii indicating myocarditis ; Ba. swallow impossible—choking with spilling of barium into the trachea ; skin biopsy, epidermal inflammatory changes present ; the corium showed hyaline degeneration of collagen and dilated vessels with a perivascular round cell infiltrate; muscle sections showed patchy degenerative changes.

Treatment with P.A.B.A. produced no improvement and muscular weakness and wasting progressed with loss of tendon reflexes. He became progressively more emaciated and at his own request in August, 1950, he returned home, where he shortly died. No post-mortem examination was carried out.

A study of these and other published records reveals several interesting features :

(*a*) Unlike adult acanthosis nigricans which is usually associated with intra-abdominal cancer, this apparent " dermatomyositis " may be found with neoplasm, of any organ or tissue. Reports include tumours of breast (Behrman and Forman, 1939), the ovary (Brunner and Lobraico, 1951) the uterus (Duverne, Bonnayme and Mounier, 1954), the larynx (Borda, 1953), the lung (Quiroga and Bottrick, 1950), the bronchus (Bouton, Mendoza and Ravera, 1953), the oesophagus (Jorgensen, 1944), the stomach (Lipman and Tober, 1950), the intestine (McCombs and MacMahon, 1947), the rectum (O'Leary and Waisman, 1940), the gall bladder (Wolf and Wilens, 1936), and the kidney (Cottell, 1952). More unusual reports include Hodgkin's disease (Curtis, Blaylock and Harrell, 1952) and other reticuloses (Sheldon, Young and Dyke, 1939), plasmocytoma (Jorgensen, 1944), multiple myeloma, retroperitoneal sarcoma and a tumour of the parotid (Curtis, Blaylock and Harrell, 1952). O'Leary (1949) quotes also two instances of Cushing's syndrome suggestive of adrenal tumour and Sunde (1949) has recently recorded a case in a girl of twelve with a chromophobe adenoma of the hypophysis. The presence of metastases is frequently, but not constantly recorded.

(*b*) There seems to be no doubt that in all instances the neoplastic process has preceded the onset of cutaneous and muscular changes though the recognition of the latter has very often preceded the detection of the former, which may only be discovered post mortem. The dermatomyositic process appears also undoubtedy to hasten the end.

(*c*) In several instances (Brunner and Lobraico, 1951 ; Curtis Blaylock and Harrell, 1952 ; Duverne, Bonnayme and Mounier, 1954), Bezecny's (1935) observation of remission with successful treatment of the neoplasm has been confirmed permanently or temporarily with relapse (Curtis, Blaylock and Harrell, 1952 ; Dostrovsky and Sagher, 1946), Lipman and Tober (1950), on the appearance of metastases.

(*d*) Though several authors have recorded cases of association of neoplasm with other collagen diseases such as scleroderma (Curtis, Blaylock and Harrell, 1952 ; Lipman and Tober, 1950) and systemic lupus erythematosus (Lansbury, 1953) the reports available do not exclude a diagnosis of " dermatomyositis " of this type. In my opinion, it is just this association with neoplasm which separates this symptom-complex from dermatomyositis of the Petges-Clejat type and from progressive systemic sclerosis.

From the clinical aspect the main distinguishing features appear to be the onset in the relevant age-group of a relatively acute illness characterised by erythematous skin lesions which very closely, in appearance, distribution and even histology, mimic those of systemic lupus erythematosus but for the very marked oedema which is localised to the sites of the rash. Though it may have been wisdom after the event, in Case 1 exposure to strong sunlight had briefly preceded the appearance of the rash. In prolonged cases, as in Case 2, the rash may settle to a more chronic pattern of poikiloderma. Muscle involvement which appears to be more of the shoulder and pelvic girdles and the proximal rather than the distal limb muscles advances from moderate swelling with tenderness and pain on movement to an atrophic paresis. It may be significant that tendon reflexes disappear—perhaps an indication of nerve involvement—and there are recorded examples of diminished peripheral sensation (Berg, 1951). Involvement of the myocardium qua muscle appears to be found frequently as in Case No. 3 of this series. Any other visceral lesions (pleurisy, ascites, etc.) are attributable to the neoplasm or to metastases. The finding of albuminuria or haematuria, for example, would invite relevant investigation for a renal or visceral growth. Unless the course of the illness is altered by successful treatment of the neoplasm, death follows quickly.

Biopsy and post-mortem sections of skin and muscle show non-specific changes of an inflammatory nature in the former and of focal disintegration or degeneration in the latter. Wainger and Lever (1949) describe connective tissue damage in the myocardium which appeared to increase the degeneration of muscle fibres. Some of the visceral sections of Cases 1 and 2 do suggest a more general involvement of connective tissue and the walls of smaller blood vessels in a widespread process of fibrinoid degeneration type. Reports by others stress particularly the vascular lesions found post mortem. McCombs and MacMahon (1947) found widespread lesions varying from " acute necrosis and chronic sclerosing oedema of all layers to healed lesions with intimal and adventitial scarring ".

Theories on the basic factors of this association can only be speculative. Biopsy and post-mortem reports have shown nothing to suggest a mechanical dissemination of tumour cells in the skin and muscles. Consideration of comparable recorded entities does provide a possible line of elucidation of this problem. A number of authors have described various forms of nervous degeneration in association with malignant disease which is most commonly but not always of bronchogenic type. Denny-Brown (1948) has published cases of this type with sensory neuropathy and myopathy which he has claimed to be due to primary simple degeneration of dorsal root ganglion cells associated with a primary degeneration of muscles. He presumed the cause to be a metabolic disorder related to the tumour cells, the changes being similar to changes seen in animals in pantothenic acid and vitamin E deficiency states. Henson (1953) has described eight such cases with a lower motor neurone atrophic paresis affecting mainly proximal muscle groups, though some showed a pseudobulbar palsy with dysphagia and dyarthria and others ptosis or diplopia. Hart (1954) has recorded two similar instances—one with a carcinoma of the breast and the other of the antrum—a male patient who incidentally showed poikilodermatous patches on the face, neck and arms. Both these improved greatly with treatment of the neoplasm though credit was given to the administration of vitamin B complex—especially pyridoxine—and a hypothesis of competition by tumour cells for this vitamin was postulated. One must assert that the skin lesions in this dermatomyositic illness are not those of frank pellagra. Lennox and Pritchard (1950) also described a series of cases, some of whom showed the proximal motor neuropathy without significant sensory change. Histological examination is such cases has shown patchy demyelination in the cord and peripheral nerves. I believe that there may well be a close relationship between these neuropathies and this type of "dermatomyositis" and would suggest histopathological examination of post-mortem cord and nerve specimens in any future cases.

It is tempting to suggest therefore that the pathogenesis of this dermatomyositic complex lies in the growth factor or invasive potential of the neoplastic process—that by competing with normal connective tissue or muscle or nerve cells for some essential nutritional factor, or perhaps by flooding the circulation with an enzymatic spreading factor like hyaluronidase to assist invasion it might provoke widespread alteration and degeneration of connective tissue ground substance, and related mesenchymal derivatives such as muscle or nerve cells. Alternatively an antigenic substance from the tumour or its breakdown products might first sensitise and then provoke an hyperimmune response, particularly in the smaller blood vessels, with secondary degenerative effects on connective tissue muscles and nerves.

The importance of awareness and recognition of this dermatomyositic symptom-complex lies in the obviously vital necessity in these cases for an exhaustive search for a neoplasm which may still be amenable to operative or radiation therapy.

### SUMMARY.

(1) These examples are presented of the association of a dermatomyositic symptom-complex with malignant disease.

(2) It is suggested that this symptom complex which is an entity separate from true chronic progressive dermatomyositis may be closely related to the published cases of nervous degeneration in association with malignant disease and, like these, may originate from a competition by tumour cells for some essential nutritional factor.

My thanks are due to Dr. T. E. Anderson for helpful advice and for permission to publish Cases 2 and 3.

### REFERENCES.

Behrman, S. and Forman, L.—(1939) *Proc. Roy. Soc. Med.*, **32**, 1575.
Berg, R. L.—(1951) *New Engl. J. Med.*, **245**, 822.
Bezecny, R.—(1935) *Arch. Derm. Syph., Wien*, **171**, 242.
Borda, J. M.—(1953) *Rev. Asoc. méd. argent.*, **67**, 755.
Bouton, J., Mendoza, D. and Ravera, J. J.—(1953) *Torax*, **2**, 47.
Brunner, M. J. and Lobraico, R.—(1951) *Ann. intern. Med.*, **34**, 1269.
Cottell, C. E.—(1952) *Amer. J. med. Sci.*, **224**, 160.
Curtis, A. C., Blaylock, H. C. and Harrell, E. R., Jr.—(1952) *J. Amer. med. Ass.*, **150**, 844.
Denny-Brown, D.—(1948) *J. Neurol. Psychiat.*, **11**, 73.
Dostrovsky, A. and Sagher, F.—(1946) *Brit. J. Derm.*, **58**, 52.
Duverne, J., Bonnayme, R. and Mounier, R.—(1954) *J. Méd., Lyons*, **35**, 601.
Hart, P. L. de V.—(1954) *Brit. med. J.*, i, 606.
Henson, R. A.—(1953) *Proc. Roy. Soc. Med.*, **46**, 859.
Jorgensen, K. S.—(1944) *Acta. Path. microbiol., scand.*, **21**, fasc iv.
Lansbury, J.—(1953) *Ann. Rheum. Dis.*, **12**, 301.
Lennox, B. and Pritchard, S.—(1950) *Quart. J. Med.*, **19**, 97.
Lipman, M. P. and Tober, J. N.—(1950) *Gastroenterology*, **16**, 188.

McCombs, R. P. and MacMahon, H. E.—(1947) *Med. Clin. N. Amer.*, **31**, 1148.
O'Leary, P. A.—(1949) *Ibid.*, **33**, 28.
*Idem* and Waisman, M.—(1940) *Arch. Derm. Syph., Chicago*, **41**, 1001.
Petges, G. and Clejat, C.—(1906) *Ann. Derm. Syph., Paris*, **7**, 550.
Quiroga, M. I. and Bottrick, A.—(1950) *J. Amer. med. Ass.* (*Abstracts*), **142**, 1028.
Sheldon, J. H., Young, F. and Dyke, S. C.—(1939) *Lancet*, i, 82.
Sunde, H.—(1949) *Acta paediatr., Stockh.*, **37**, 287.
Unverreicht, H.—(1891) *Dtsch. med. Wschr.*, **17**, 41.
Wainger, C. L. and Lever, W. F.—(1949) *Arch. Derm. Syph., Chicago*, **59**, 196.
Wolf, A. and Wilens, S. L.—(1936) *Amer. J. Path.*, **12**, 239.